# The spectrum of sickle cell disease

**DOI:** 10.1002/ajh.27494

**Published:** 2024-10-01

**Authors:** Barbara J. Bain

**Affiliations:** ^1^ Centre for Haematology, St Mary's Hospital Campus of Imperial College Faculty of Medicine, St Mary's Hospital London UK

Sickle cell disease is a somewhat ambiguous term, sometimes used as a synonym for sickle cell anemia and sometimes as a generic term, encompassing also compound heterozygous states characterized by clinicopathological features that result from sickle cell formation. There are at least 13 compound heterozygous states of relevance, the most common being sickle cell/β thalassemia, sickle cell/hemoglobin C disease, and sickle cell/hemoglobin D disease. The thalassemia may be β^0^ or β^+^. The hemoglobin D is specifically hemoglobin D‐Punjab/D‐Los Angeles. In addition, rarely the phenotype of sickle cell disease results from heterozygosity for a variant hemoglobin with a second mutation in a β^S^ gene, specifically S‐Oman, S‐Antilles, and hemoglobin Jamaica Plain.

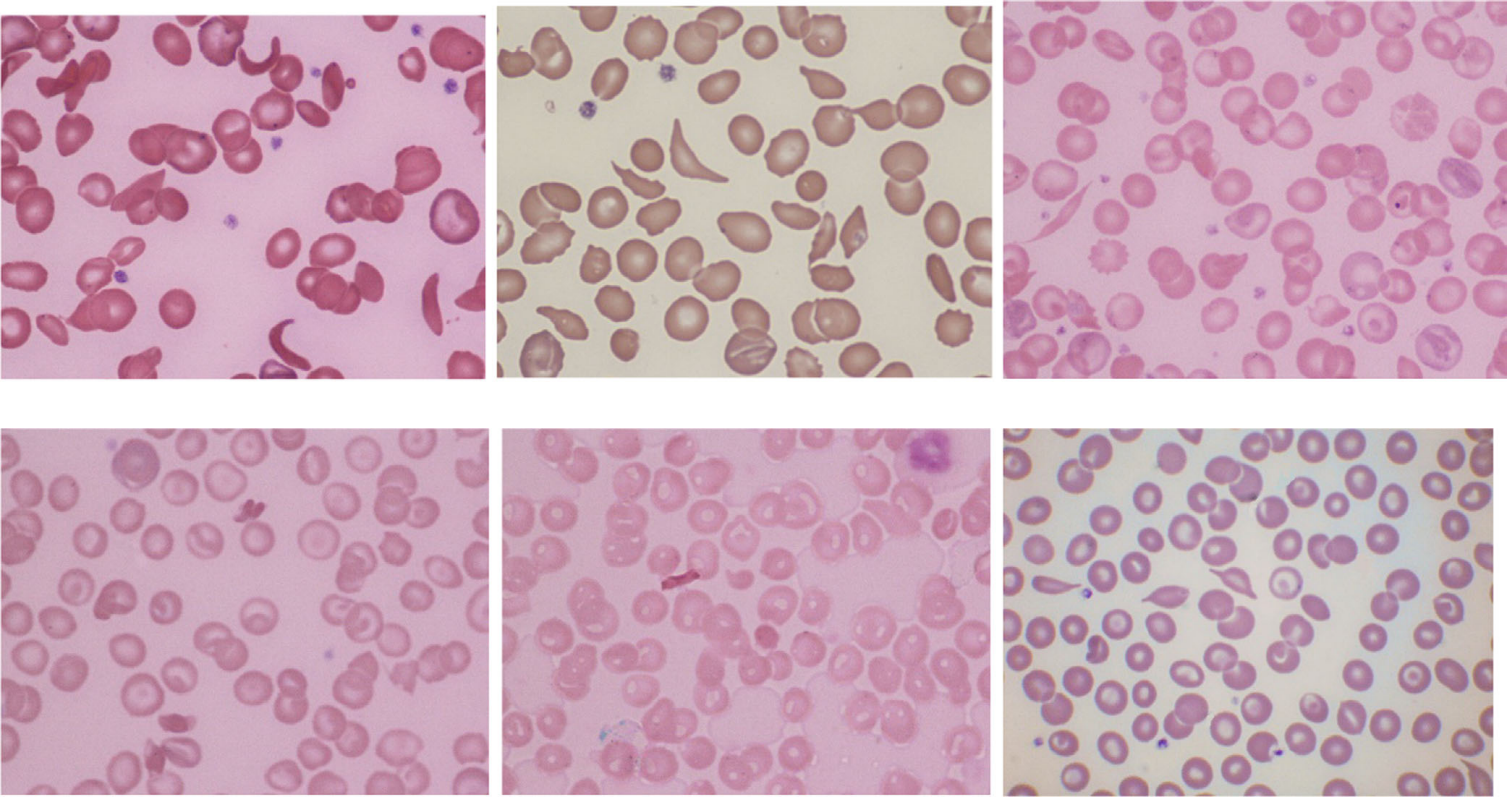

The hemoglobin concentration (Hb) in sickle cell anemia is typically 60–100 g/L with the blood film (top left, all images May–Grünwald–Giemsa, ×100 objective) showing sickle cells boat‐shaped cells, target cells, features of hyposplenism (Howell–Jolly bodies, Pappenheimer bodies, large platelets), nucleated red blood cells, polychromasia and sometimes irregularly contracted cells or linear fragments. Some compound heterozygous states, for example, SD (top center), have hematological features very similar to those of sickle cell anemia.^1^ Others differ. Microcytosis and hypochromia are additional features in sickle cell/β thalassemia (top right). Sickle cell/hemoglobin C disease also differs. The Hb tends to be higher and the blood film may show distinctive SC poikilocytes when the crystallization of hemoglobin C and polymerization of hemoglobin S occur in the same cell^2^; hemoglobin C crystals may also be present (bottom left and center). A distinctive poikilocyte is also associated with hemoglobin S‐Oman in heterozygotes (bottom right), homozygotes, and compound heterozygotes; these poikilocytes have been referred to as Napolean hat cells.

The different types of sickle cell disease differ in their clinicopathological characteristics and disease outcomes. It is, therefore, important that the heterogeneity is recognized and that articles in scientific journals and presentations at congresses make clear in what sense the term “sickle cell disease” is being used.^3^

## CONFLICT OF INTEREST STATEMENT

The author declares no conflict of interest.
